# Development and validation of a comprehensive tool for measuring multidimensional peritraumatic experience

**DOI:** 10.1590/0102-311XEN062225

**Published:** 2025-12-01

**Authors:** Evandro Silva Freire Coutinho, Michael Eduardo Reichenheim, Thaiza Dutra Gomes de Carvalho, Leticia Rocha Pereira, Ivan Figueira, Sérgio Baxter Andreoli, Maria Inês Quintana, Jair de Jesus Mari

**Affiliations:** 1 Instituto de Medicina Social Hesio Cordeiro, Universidade do Estado do Rio de Janeiro, Rio de Janeiro, Brasil.; 2 Escola Nacional de Saúde Pública Sergio Arouca, Fundação Oswaldo Cruz, Rio de Janeiro, Brasil.; 3 Serviço de Medicina Interna, Universidade do Estado do Rio de Janeiro, Rio de Janeiro, Brasil.; 4 Instituto de Psiquiatria, Universidade Federal do Rio de Janeiro, Rio de Janeiro, Brasil.; 5 Departamento de Psiquiatria, Universidade Federal de São Paulo, São Paulo, Brasil.; 6 Universidade Católica de Santos, Santos, Brasil.

**Keywords:** Tonic Immobility Response, Dissociative Disorders, Panic, Psychometrics, Questionnaires, Resposta de Imobilidade Tônica, Transtornos Dissociativos, Pânico, Psicometria, Questionários, Pérdida de Tono Postural, Trastornos Disociativos, Pánico, Psicometría, Cuestionarios

## Abstract

Despite available instruments to assess peritraumatic reactions - such as tonic immobility, dissociation, and panic reactions - content overlap remains an issue. To date, no comprehensive tool integrates these dimensions into a single valid instrument. This study consolidates items from various sources to create a standalone solution, employing both qualitative and quantitative approaches. At several stages, experts examined potential content overlaps and redundancies in item components, supported by psychometric assessments using exploratory structural equation models and confirmatory factor analysis. The 29-item instrument exhibits robust configural and metric properties, including unique items per factor, high reliability, absence of content redundancy, and convergent and discriminant factorial validity. The provisionally named *Peritraumatic Response Questionnaire* (PTRQ) seems suitable for use in epidemiological studies in its current state. Further refinement may enhance its comprehensiveness and suitability for broader applications. Thus, we propose this instrument for assessing peritraumatic experiences, with potential for future enhancement by continued research.

## Abstract (pt)

Apesar dos instrumentos disponíveis para mensurar reações peritraumáticas, como imobilidade tônica, dissociação e reações de pânico, a sobreposição do conteúdo permanece problemática. Até o momento, nenhuma ferramenta abrangente integra essas dimensões em um único instrumento válido. Este estudo une itens de diversas fontes para criar uma solução singular. Para tanto, empregamos abordagens tanto qualitativas quanto quantitativas. Em várias etapas, especialistas examinaram potenciais sobreposições e redundâncias de conteúdo nos componentes dos itens, apoiados por avaliações psicométricas utilizando modelos exploratórios de equações estruturais e modelos analíticos fatoriais confirmatórios. O instrumento de 29 itens apresenta propriedades robustas de mensuração configuracional e métrica, incluindo itens únicos por fator, alta confiabilidade e ausência de redundância de conteúdo, bem como validade fatorial convergente e discriminante. O *Questionário de Resposta Peritraumática* (QRPT), provisoriamente denominado, parece adequado para uso em estudos epidemiológicos em seu estado atual. Refinamentos adicionais podem aumentar sua abrangência e adequação para aplicações mais amplas. Sendo assim, propomos este instrumento para avaliar experiências peritraumáticas, com potencial para aprimoramento futuro por meio de pesquisas contínuas.

## Abstract (es)

A pesar de que existen instrumentos disponibles para medir las reacciones peritraumáticas, como la inmovilidad tónica, la disociación y las reacciones de pánico, la superposición de contenidos sigue siendo un problema. Hasta la fecha, no existe ninguna herramienta completa que integre estas dimensiones en un único instrumento válido. Este estudio consolida elementos de diversas fuentes para crear una solución independiente. Para ello, empleamos enfoques tanto cualitativos como cuantitativos. En varias etapas, los expertos examinaron posibles superposiciones y redundancias en el contenido de los componentes de los ítems, con el apoyo de evaluaciones psicométricas que utilizaron modelos exploratorios de ecuaciones estructurales y modelos confirmatorios de análisis factorial. El instrumento de 29 ítems presenta sólidas propiedades de medición de configuraciones y métricas, incluyendo ítems únicos por factor, alta fiabilidad, ausencia de redundancia de contenido y validez factorial convergente y discriminante. El cuestionario denominado provisionalmente *Cuestionario de Respuesta Peritraumática* (CRP) parece adecuado para estudios epidemiológicos en su formato actual. Un mayor perfeccionamiento podría mejorar su exhaustividad y adecuación para aplicaciones más amplias. Por lo tanto, proponemos este instrumento para evaluar las experiencias peritraumáticas, con potencial para futuras mejoras a través de la investigación continua.

## Introduction

Studies from different countries report that around 90% of the population experience potential traumatic events throughout their lifetimes ^1^. Trauma affects victims differently, both in their responses and in the intensity of their experiences during or immediately after the event ^2^.

The defense cascade theory is perhaps the most well-structured to explain these reactions resulting from involuntary physiological responses activated by the release of neurotransmitters in threatening situations. Schauer & Elbert ^3^ suggest a sequence of six responses (Freeze-Flight-Fight-Fright-Flag-Faint), which evolve gradually based on the victim’s evaluation of threat proximity, severity, and their capacity for defense.

According to a theory developed based on animal behavior, the first stage of the defensive cascade (Freeze) enhances the chance that prey will go unnoticed by a predator. If the threat persists, there is a more intense and prolonged activation of the sympathetic division of the autonomic nervous system, followed by the Flight and Fight responses. If escape is impossible, the prey resorts to fighting ^3^. Sympathetic predominance during the traumatic experience leads to hyperarousal symptoms, including increased heart and respiratory rates, elevated blood pressure, vasoconstriction, and general fear responses, such as panic-like reactions. A shift occurs when the parasympathetic nervous system becomes dominant, triggering the Flag and Faint stages. Under parasympathetic dominance, dissociative manifestations prevail, while tonic immobility reactions occur during the transition phase when both systems are concurrently activated ^3^. Notably, trauma response patterns are individualized and do not necessarily involve all six stages of the defensive cascade.

Dissociation, tonic immobility, and physical manifestations of panic are the most studied peritraumatic reactions. Dissociation encompasses a variety of responses that disrupt the balanced and integrated functions of consciousness, memory, identity, and environmental perception ^4^ ^,^ ^5^.

Tonic immobility is a temporary catatonic state marked by reversible motor inhibition, muscular hypertonicity, analgesia, and a relative lack of response to external stimuli ^6^. Initially observed in animals, studies in humans first involved women victims of sexual abuse and rape, who reported paralysis and inability to scream during the assault ^7^. Tonic immobility may be considered an evolutionary adaptation, increasing the likelihood of survival by reducing the probability that predators attack or kill immobile prey ^3^. However, its occurrence in humans may decrease chances of success in some contexts, such as immediate evacuation during a disaster or a car crash ^8^.

Panic attacks are abrupt onsets of intense fear or discomfort that peak within minutes and are accompanied by physical and cognitive symptoms ^9^. Common physical reactions - such as tachycardia, chest pain, tremors, nausea, dizziness, and shortness of breath - are due to sympathetic and parasympathetic system activation during the traumatic event ^3^. Studies show that about half of victims report undergoing such reactions during a traumatic event ^10^.

These reactions, occurring during or shortly after a traumatic event, have been linked to several adverse medium- and long-term outcomes, primarily on mental health. Studies have suggested associations between these reactions and depression ^11^ as well as post-traumatic stress disorder (PTSD) ^5^ ^,^ ^11^ ^,^ ^12^ ^,^ ^13^ ^,^ ^14^. Regarding the latter, peritraumatic reactions have also been linked to greater symptom severity and poorer treatment outcomes ^12^ ^,^ ^13^. However, the literature remains somewhat inconsistent regarding which types or combinations of reactions are most strongly associated with PTSD and its progression. Some authors propose that dissociation is the strongest PTSD predictor ^13^ ^,^ ^15^, while others argue that tonic immobility is more important for both the development and severity of the disorder ^12^. The onset of tonic immobility has also been implicated in poorer responses to pharmacological treatment ^12^. Massazza et al. ^16^ evaluated the predictive capacity of different peritraumatic reactions on four PTSD symptom clusters in earthquake survivors. They found that somatoform dissociation was related to all four symptoms - intrusion, avoidance, negative alterations in cognition and mood, and hyperexcitability - whereas tonic immobility was linked only to the first two. It has also been suggested that dissociation may mediate the relationship between panic reactions and PTSD ^17^.

Several measurement scales have been proposed to retrieve, via memory, the psychological, physiological, and somatic reactions occurring during or shortly after a traumatic event. These instruments were initially designed to target each type of peritraumatic reaction, with items primarily targeting core features of each reaction. However, a closer inspection shows some content overlap. For instance, the item “trembling or shaking” appears in both the *DSM-based Physical Reactions Scale* ^10^ and the *Tonic Immobility Scale* (TIS) ^18^. Similarly, the latter includes two items (“feeling disconnected from oneself, as if leaving the body” and “feeling distant from the situation, as if going elsewhere”), which address typically dissociative manifestations ^3^.

The defense cascade theory gathers several elements. It supports the hypothesis that tonic immobility, dissociation, and panic reactions are distinct facets of a multidimensional construct operating during or immediately after trauma - the peritraumatic reaction. To the best of our knowledge, the existing literature does not provide a comprehensive measurement tool that captures the various manifestations of peritraumatic experiences across multiple dimensions in an integrated manner. An instrument with these characteristics and robust psychometric properties would be valuable to investigate the interplay among these dimensions and their impact on both physical and mental health.

To develop an integrated, comprehensive, and high-quality measurement instrument, we examined three commonly used tools or item sets, addressing potential overlaps and redundancies in content and item components, as well as psychometrically testing several favorable properties in the final selected set. 

## Materials and methods

### Data source - the original project

This investigation used data from a comprehensive research project titled *The Impact of Violence on the Mental Health of the Brazilian Population* (Millennium Institute/CNPq Project). The project aimed to evaluate the impact of urban violence on mental health in the two largest cities of Brazil, São Paulo and Rio de Janeiro, based on representative samples of individuals aged 15 to 75. Data collection occurred between June 2007 and January 2008. Sampling was stratified and conducted in multiple stages. In the first stage, areas of both cities were classified based on their homicide rates and subsequently grouped into seven strata: ≤ 10 homicides/100,000 inhabitants; 10.01 to 20; 20.01 to 30; 30.01 to 40; 40.01 to 50; 50.01 to 60; and > 60 homicides/100,000 inhabitants. In the second stage, census tracts of each stratum were mapped and randomly selected, with the number of tracts ranging from four to 18 depending on the population size of the stratum. In the third stage, 43 households were randomly selected per census tract in São Paulo and 30 in Rio de Janeiro. Residents aged 15 to 75 years old were listed, and one was selected using the Kish method. For additional information, see Andreoli et al. ^19^.

Respondents completed a questionnaire including 11 traumatic events from the *Composite International Diagnostic Interview* (CIDI 2.1) ^20^. Another 21 events reflecting experiences common in urban centers were added to the survey. Of the 3,744 respondents in both cities, 3,239 (86%) reported at least one traumatic event during their lifetime ^1^. The “worst” self-reported traumatic event was used to evaluate peritraumatic reactions. Fifteen cases were excluded due to ambiguous responses. The final sample consisted of 3,224 participants, with 2,149 from São Paulo and 1,075 from Rio de Janeiro.

### Development of the instrument prototype

A prototype instrument for measuring peritraumatic reactions was developed in three stages. The first stage involved the theoretical construction of the instrument and was essentially qualitative. In the second stage, additional qualitative assessments were conducted to discuss the findings from initial explorations concerning the configurational structure of the instrument. Based on these assessments, a set of items was selected for further examination in the third and final stage, which primarily focused on the metric structure of the instrument. The procedures undertaken in the three stages are described in detail as follows.

#### Stage 1 - theoretical construction of the instrument

In this stage, the first prototype of the instrument (Prototype 1) was developed. The initial selection of components was based on four sets of items. The first two comprised the TIS ^18^ and the *Peritraumatic Dissociative Experiences Questionnaire* (PDEQ) ^4^, representing the peritraumatic tonic immobility and peritraumatic dissociation dimensions. These scales had previously been adapted into Brazilian Portuguese for the original project. Their items were kept in their original formats, either as questions requiring responses or as affirmative statements to be endorsed.

The third component included ten of 13 DSM-IV criteria for panic attacks ^9^, specifically adapted to Brazilian Portuguese by psychiatry specialists for the background project. These items contained four ordinal response options, consistent with the original project (the Millennium Institute/CNPq Project). This dimension is henceforth referred to as peritraumatic physical reactions. The label was chosen to group reactions that occur in response to a traumatic event, distinguishing them from another set of symptoms typical of panic disorder, which are unexpected and recurrent.

During this phase, four specialists with experience in PTSD (two epidemiologists and two psychiatrists) evaluated not only items from the three previously mentioned sets but also six additional items developed for the background project. These items were included due to their relevance for peritraumatic experiences and their alignment with the defensive cascade model ^3^. This fourth component consists of the items res_it1, res_it2, res_it3, res_pd1, res_ppr1, and res_ppr2, which are presented in Box 1 along with the other items.

**Box 1:** Original items from the *Tonic Immobility Scale* (TIS), *Peritraumatic Dissociative Experience Questionnaire* (PDEQ), and peritraumatic physical reactions, as well as additional items used in the background project.

TIS *		PDEQ **		PERITRAUMATIC PHYSICAL REACTIONS *** ^#^	
tis1	Rate the degree to which you froze or felt paralyzed during your most recent experience	pdeq1	I had moments of losing track of what was going on - I “blanked out” or “spaced out” or in some way felt I was not part of what was going on	ppr1	Sensations of shortness of breath or smothering
tis2	Rate the degree to which you were unable to move, although unrestrained	pdeq2	I found that I was on “automatic pilot” - I ended up doing things I later realized I had not actively decided to do	ppr2	Feeling dizzy, unsteady, lightheaded, or faint
tis3	Rate the degree to which your body was trembling/shaking during the event	pdeq3	My sense of time changed - things seemed to be happening in slow motion	ppr3	Palpitations, pounding heart, or accelerated heart rate
tis4	Rate the degree to which you were unable to call out or scream during the event	pdeq4	What was happening seemed unreal to me, like I was in a dream or watching a movie or play	ppr4	Trembling or shaking
tis5	Rate the degree to which you felt numb or no pain during the event	pdeq5	I felt as though I were a spectator watching what was happening to me, as if I were floating above the scene or observing it as an outsider	ppr5	Sweating
tis6	Rate the degree to which you felt cold during the event	pdeq6	There were moments when my sense of my own body seemed distorted or changed, I felt disconnected from my own body, or that it was unusually large or small	ppr6	Nausea or abdominal distress
tis7	Rate the extent to which you felt feelings of fear/panic during the event	pdeq7	I felt as though things that were actually happening to others were happening to me, like I was being trapped when I really was not	ppr7	Paresthesias (numbness or tingling sensations)
tis8	Rate the extent to which you feared for your life or felt as though you were going to die	pdeq8	I was surprised to find out afterward that a lot of things had happened at the time that I was not aware of, especially things I ordinarily would have noticed	ppr8	Chills or hot flushes
tis9	Rate the extent to which you felt detached from yourself during the event	pdeq9	I felt confused; that is, there were moments when I had difficulty making sense of what was happening	ppr9	Feeling of choking
tis10	Rate the extent to which you felt detached from what was going on around you during the event	pdeq10	I felt disoriented; that is, there were moments when I felt uncertain about where I was or what time it was	prs10	Chest pain or discomfort
ADDITIONAL ITEMS SUGGESTED BY THE EXPERTS					
res_it1	[Tell us how much you felt unable to escape even though you wanted to]	res_pd1	[I felt “numbed” or emotionless]	res_ppr1	[Difficulty controlling your bladder or bowel]
res_it2	[Say how guilty or ashamed you felt after the event]			res_ppr2	[If passed out during the trauma]
res_it3	[Say how much you can remember the details of the event]				

* Fuse et al. ^18^;

** Marmar et al. ^4^;

*** American Psychiatric Association ^9^;

^#^ Items from the “Panic Attack Criteria” of the 4^th^ edition of the *Diagnostic and Statistical Manual of Mental Disorders* (DSM-IV) ^9^.

A total of three in-person meetings were held with the experts, focusing on the theoretical concept of peritraumatic reactions proposed by Schauer & Elbert ^3^ regarding the defense cascade. During these meetings, the experts evaluated content validity of the initial 36 items and allocated them to the three proposed dimensions. They also discussed potential item reallocations between dimensions based on semantic content, regardless of their original scale. These meetings resulted in Prototype 1.

#### Stage 2 - exploratory evaluation of the configural structure

Prototype 1 underwent an exploratory evaluation of its configural properties, based on the three-dimensional structure of the PR construct suggested by the experts. To empirically test this theoretical proposition, we performed a principal component analysis (PCA) to obtain eigenvalues. Results were evaluated using the Kaiser-Guttman rule and the scree test, along with a graphical visualization of the related scree plots ^21^. Moreover, we fitted exploratory structural equation models (ESEM) to inspect both the a priori stipulated trifactorial structure and the structure suggested by the PCA ^22^. ESEMs offer advantages over traditional exploratory models, as they enable the inspection of residual correlations in addition to loading profile evaluation.

During the evaluation stage, several aspects were considered. First, the proposed dimensionality from the previous stage was examined to determine the optimal number of factors. Second, the theoretical-empirical congruence of the items was evaluated by examining their assumed alignment with their respective dimensions. Finally, the factorial specificity of each item was explored to clarify factorial loadings. Items were considered non-specific if they had two or more loadings above 0.30 in distinct factors, and pertinence violations occurred if an item mostly loaded on a factor other than the one theorized, provided no cross-loadings were observed ^21^. Exploratory findings and expert evaluations led to the refinement of the initial prototype, resulting in a new proposal (Prototype 2).

#### Stage 3 - evaluation of the metric structure via confirmatory analysis

During this stage, models were adjusted based on the dimensional structure of Prototype 2 using ESEM and confirmatory factor analyses (CFA). The evaluation of the metric structure included exploring the following properties: (a) item reliability, examining the magnitude of factor loadings and residuals; (b) identification of content redundancies by the presence of possible residual correlations; (c) factor-based discriminant validity; and (d) factor-based convergent validity ^23^.

Item reliability was considered insufficient if factor loadings were below 0.5 or residual variances (uniqueness) exceeded 0.70. Therefore, only items meeting these thresholds were considered suitable for the factor. When residual correlations exceeded 0.30 and had theoretical support, items were considered redundant, provided they were semantically similar ^24^. Decisions regarding insufficient reliability or redundancy were based on findings from at least one of the two cities analyzed.

In addition to examining the magnitude of factor loadings, modification indices (a univariate Lagrange multiplier) and expected parameter changes were assessed for all items in samples from São Paulo and Rio de Janeiro ^21^. Modification indices indicates the expected decrease in the chi-square statistic of the model if the corresponding parameter were freely estimated in the tested model ^25^. Items showing issues in metric properties, such as low reliability (factor loadings), were reviewed on a case-by-case basis in meetings with specialists to determine whether they should be excluded or retained in subsequent interim analyses.

To formally evaluate factor-based discriminant validity, the square root of the average variance extracted (AVE) of each fator $\left(\sqrt{\rho_{ave\left(f_{k}\right)}}\right)$ was compared with their respective factorial correlations $\left(\phi_{f_{\left(k\right)}↔f_{\left(\neq k\right)}}\right)$ ^21^ ^,^ ^26^. AVE reflects the proportion of variance in a factor captured by the observed items relative to the variance attributed to measurement error, representing the portion not explained by the latent factor. Values range from 0 to 1. To support factor-based discriminant validity, the square root of AVE for a given factor must exceed its correlations with all other factors in the model $\left(\sqrt{\rho_{ave\left(f_{g}\right)}}>\Phi_{\left(f_{\left(g\right)},f_{\left(h\neq g\right)}\right)}\right)$ . Formally testing $\sqrt{\rho_{ave\left(f_{g}\right)}}-\Phi_{\left(f_{\left(g\right)},f_{\left(h\neq g\right)}\right)}$ , a statistically significant positive result indicates support for factor-based discriminant validity (i.e., non-violation), while a negative result suggests rejection. A conservative approach was adopted, considering only significantly negative differences to indicate factor-based discriminant validity rejection. Additionally, 95% confidence intervals were estimated using bootstrap (1,000 replicates). AVE was also used to assess factor-based convergent validity, which is admissible if above 0.50, indicating that at least half of the variance in a measure is attributable to the underlying latent trait ^21^ ^,^ ^26^.

We used the robust diagonally weighted least squares estimator, as it is appropriate for polychotomous and ordinal items ^27^. Model fit was assessed using three indices: root mean square error of approximation (RMSEA), comparative fit index (CFI), and Tucker-Lewis index (TLI). RMSEA values below 0.06 indicate acceptable fit, while values above 0.10 suggest clear inadequacy and warrant model rejection. CFI and TLI range from 0 to 1, with values greater than 0.95 indicating adequate fit ^21^.

Descriptive analyses were performed using Stata version 16 (https://www.stata.com), while the main analyses were conducted using Mplus version 8 (https://www.statmodel.com/). Complex sampling procedures, including stratification and clustering, were considered throughout ^28^.

### Ethical issues

This study was approved by the Research Ethics Committee of the Federal University of São Paulo (process n. 1369-1304), in accordance with the principles of the *Declaration of Helsinki*.

## Results

The sample was slightly skewed toward women in both cities, with 56.8% in São Paulo and 55.7% in Rio de Janeiro. In the former, the mean age was 40.0 years for women and 39.0 years for men. In the latter, the corresponding averages were 43.6 and 40.8 years. The most frequently reported traumas in São Paulo were the death of a loved one (27.3%), direct exposure to urban violence (19.9%), and witnessing urban violence (9.5%). In Rio de Janeiro, these three categories were the most prevalent, with frequencies of 30.5%, 21.4%, and 11%, respectively.

During the first stage of the process (development of Prototype 1), two items from the original study - res_it2 (feeling guilt/shame) and res_it3 (remembering details) - were excluded by the experts. The remaining four items specifically developed for the original study were allocated to their respective dimensions. Specifically, res_it1 (unable to escape) was assigned to the peritraumatic tonic immobility dimension, res_pd1 (numbed) to the peritraumatic dissociation dimension, and res_ppr1 (control bladder or bowels) and res_ppr2 (fainting) to the peritraumatic physical reactions dimension.

During the theoretical-conceptual stage of allocating items from three instruments, two TIS items related to dissociative symptoms (tis9: disconnected from oneself; and tis10: distant from the situation) were moved to the peritraumatic dissociation dimension. Items addressing intense fear (tis7: fear or panic; and tis8: thinking they were going to die) were kept in the peritraumatic tonic immobility dimension despite being common in panic attacks during a trauma.

In the second stage, a preliminary PCA showed four eigenvalues above 1.0 in both cities - São Paulo: eig_(f1)_ = 17.8; eig_(f2)_ = 2.58; eig_(f3)_ = 1.88; eig_(f4)_ = 1.24; Rio de Janeiro: (eig_(f1)_ = 18.9; eig_(f2)_ = 2.61; eig_(f3)_ = 1.67; eig_(f4)_ = 1.22. While the scree test supported a four-factor structure, ESEM revealed that most items in the fourth factor had loadings below 0.30. Only three items showed loadings in the range of 0.40-0.50: λ_tis3_ = 0.402 and 0.348, λ_ppr3_ = 0.400 and 0.408, and λ_ppr4_ = 0.431 and 0.436 in São Paulo and Rio de Janeiro, respectively. Theoretical aspects ultimately led to the fourth dimension being excluded from subsequent analyses.

Table 1 presents the findings of the three-factor ESEM. Some items in the São Paulo (tis6, tis9, and res_ppr2) and Rio de Janeiro (tis6, tis8, pdeq2, tis10, and res_ppr2) samples had factor loadings below 0.5 on their intended factors. There were also cross-loadings involving tis3, tis7, tis8, tis9, tis10, ppr3, and ppr4 items. A preliminary inspection of the modification indices obtained from ESEM suggested a residual correlation between ppr2 and res_ppr2, projecting an expected parameter changes above 0.3.

**Table 1:** Exploratory analyses of Prototype 1 of the peritraumatic reactions instrument using three-factor exploratory structural equation models (ESEM) in the São Paulo and Rio de Janeiro, Brazil, samples.

Item source	São Paulo				Rio de Janeiro			
	λ_i(f1)_ *	λ_i(f2)_ *	λ_i(f3)_ *	δ_i_ **	λ_i(f1)_ *	λ_i(f2)_ *	λ_i(f3)_ *	δ_i_ **
Peritraumatic tonic immobility								
tis1	0.728	0.192	-0.031	0.035	0.676	0.178	0.078	0.285
tis2	0.786	0.233	-0.128	0.249	0.713	0.231	-0.001	0.226
tis3	0.570	-0.012	0.313	0.398	0.616	-0.023	0.383	0.285
tis4	0.605	0.203	0.058	0.395	0.681	0.083	0.118	0.357
tis5	0.600	0.244	0.009	0.403	0.525	0.308	-0.021	0.437
tis6	0.388	0.250	0.234	0.443	0.499	0.222	0.146	0.433
tis7	0.513	0.046	0.305	0.427	0.646	0.017	0.243	0.360
tis8	0.599	-0.103	0.306	0.453	0.483	0.037	0.279	0.528
res_it1 ***	0.703	0.029	0.115	0.377	0.700	0.119	0.083	0.316
Peritraumatic dissociation								
pdeq1	0.133	0.683	0.048	0.357	0.133	0.769	-0.085	0.337
pdeq2	0.116	0.594	0.045	0.510	0.078	0.491	0.140	0.594
pdeq3	0.135	0.660	0.085	0.348	0.117	0.688	0.080	0.330
pdeq4	-0.009	0.774	-0.019	0.428	0.134	0.701	-0.003	0.371
pdeq5	-0.056	0.858	-0.014	0.331	-0.001	0.835	-0.027	0.330
pdeq6	0.105	0.743	0.102	0.222	-0.045	0.925	0.021	0.175
pdeq7	-0.023	0.808	0.026	0.340	-0.161	0.857	0.096	0.329
pdeq8	-0.052	0.869	-0.020	0.316	-0.002	0.736	0.077	0.389
pdeq9	-0.006	0.894	-0.074	0.291	0.002	0.786	0.107	0.272
pdeq10	0.033	0.860	-0.005	0.233	0.077	0.741	0.129	0.235
res_pd1 ***	0.052	0.784	0.001	0.335	0.016	0.771	0.090	0.301
tis9	0.366	0.467	0.077	0.365	0.354	0.555	-0.031	0.341
tis10	0.289	0.514	-0.024	0.505	0.355	0.465	-0.049	0.484
Peritraumatic physical reactions								
ppr1	-0.002	0.010	0.805	0.342	0.070	0.112	0.704	0.341
ppr2	0.012	0.026	0.782	0.350	0.103	0.046	0.753	0.303
ppr3	0.226	-0.122	0.697	0.425	0.332	-0.105	0.679	0.337
ppr4	0.212	-0.057	0.703	0.365	0.321	-0.074	0.707	0.273
ppr5	0.041	0.019	0.763	0.361	0.186	0.005	0.744	0.280
ppr6	-0.099	0.023	0.857	0.323	0.073	0.231	0.675	0.239
ppr7	0.019	0.000	0.837	0.282	-0.082	0.294	0.758	0.164
ppr8	0.019	0.051	0.798	0.288	0.009	0.242	0.686	0.271
ppr9	-0.049	0.067	0.876	0.196	0.072	0.186	0.697	0.261
ppr10	-0.003	0.069	0.805	0.274	-0.023	0.163	0.761	0.272
res_ppr1 ***	-0.019	0.096	0.688	0.445	-0.077	0.267	0.717	0.266
res_ppr2 ***	0.069	0.037	0.457	0.725	-0.024	0.062	0.484	0.740
RMSEA ^#^	0.022 (0.020-0.024)			0.021 (0.017-0.024)				
CFI	0.987			0.989				
TLI	0.984			0.986		

CFI: comparative fit index; RMSEA: root-mean-square error of approximation; TLI: Tucker-Lewis index.

* Cross-loadings;

** Measurement errors;

*** Items specifically designed for the background project;

^#^ 90% confidence interval.

Based on these findings, a new round of meetings was conducted to discuss three main issues: (a) insufficient factor-specificity of some items; (b) overlapping item content; and (c) problematic performance of certain items from the original scales. As a result, five items were excluded - tis6 (feeling cold), tis9 (disconnected from oneself), tis10 (distant from the situation), pdeq2 (“autopilot”), and res_ppr2 (fainting) - resulting in the creation of Prototype 2. Additional rationale for excluding or retaining these items are provided in the *Discussion* section.

Moving to the third stage, Prototype 2 underwent confirmatory factor analyses in both the São Paulo and Rio de Janeiro samples. Model fit indices were adequate, with all items exhibiting factor loadings exceeding 0.7 and residuals below 0.5. Additionally, interim diagnostics based on modification indices did not indicate residual correlations greater than 0.3. Results are presented in Table 2.

**Table 2:** Confirmatory factor analysis (CFA) of the three-dimensional structure of Prototype 2 of the peritraumatic reactions instrument in the São Paulo and Rio de Janeiro, Brazil, samples.

Item * **		São Paulo		Rio de Janeiro	
		λ_i_ ***	δ ^#^	λ_i_ ***	δ ^#^
Peritraumatic tonic immobility					
pr1	(tis1)	0.815	0.337	0.840	0.294
pr2	(tis2)	0.819	0.329	0.858	0.263
pr3	(tis3)	0.788	0.379	0.857	0.266
pr4	(tis4)	0.786	0.382	0.785	0.383
pr5	(tis5)	0.766	0.414	0.741	0.451
pr6	(tis7)	0.787	0.381	0.797	0.364
pr7	(tis8)	0.716	0.487	0.708	0.498
pr8	(res_it1) ^##^	0.764	0.416	0.800	0.361
$\sqrt{{ave}_{\left(f1\right)}}$		0.785		0.801	
Peritraumatic dissociation					
pr9	(pdeq1)	0.810	0.343	0.793	0.370
pr10	(pdeq3)	0.827	0.316	0.836	0.302
pr11	(pdeq4)	0.747	0.442	0.797	0.365
pr12	(pdeq5)	0.794	0.370	0.794	0.370
pr13	(pdeq6)	0.902	0.186	0.885	0.217
pr14	(pdeq7)	0.811	0.342	0.799	0.361
pr15	(pdeq8)	0.805	0.353	0.784	0.385
pr16	(pdeq9)	0.825	0.319	0.860	0.260
pr17	(pdeq10)	0.874	0.237	0.897	0.196
pr18	(res_pd1) ^##^	0.817	0.332	0.843	0.289
$\sqrt{{ave}_{\left(f2\right)}}$		0.837		0.838	
Peritraumatic physical reactions					
pr19	(ppr1)	0.803	0.355	0.806	0.351
pr20	(ppr2)	0.797	0.365	0.812	0.341
pr21	(ppr3)	0.762	0.420	0.809	0.346
pr22	(ppr4)	0.819	0.329	0.852	0.273
pr23	(ppr5)	0.797	0.365	0.833	0.306
pr24	(ppr6)	0.785	0.383	0.889	0.210
pr25	(ppr7)	0.839	0.297	0.903	0.184
pr26	(ppr8)	0.843	0.289	0.855	0.269
pr27	(ppr9)	0.889	0.210	0.865	0.251
pr28	(ppr10)	0.855	0.270	0.834	0.304
pr29	(res_ppr1) ^##^	0.750	0.438	0.845	0.286
$\sqrt{{ave}_{\left(f3\right)}}$		0.814		0.844	
$\emptyset_{f_{\left(1\right)}↔f_{\left(2\right)}}$		0.743		0.799	
$\emptyset_{f_{\left(1\right)}↔f_{\left(3\right)}}$		0.726		0.764	
$\emptyset_{f_{\left(2\right)}↔f_{\left(3\right)}}$		0.709		0.737	
RMSEA ^###^		0.024 (0.022-0.026)		0.023 (0.020-0.027)	
CFI		0.986		0.988	
TLI		0.985		0.988	

AVE: average variance extracted; CFI: comparative fit index; RMSEA: root-mean-square error of approximation; TLI: Tucker-Lewis index.

* “pr” stands for peritraumatic response;

** Acronym of the item according to the instrument of origin in brackets;

*** Standardized loadings;

^#^ Measurement error;

^##^ Items specifically designed for the background project (c.f., Table 1);

^###^ 90% confidence interval.

All AVE values exceeded 0.5, both in São Paulo (AVE_(f1)_ = 0.616; AVE_(f2)_ = 0.701; AVE_(f3)_ = 0.662) and Rio de Janeiro (AVE_(f1)_ = 0.642, AVE_(f2)_ = 0.702, AVE_(f3)_ = 0.713). Table 3 displays differences between AVE square roots for each factor and their corresponding factorial correlations for Prototype 2 in both cities. No AVE square roots were below zero. In São Paulo, all values were statistically significant (p < 0.05), fully supporting factor-based discriminant validity. In Rio de Janeiro, while two values related to the AVE of the first factor did not reach statistical significance, the findings still aligned with factor-based discriminant validity, as only negative and significant differences were considered indicative of a violation.

**Table 3:** Difference between the square root average variances extracted from each factor and the factorial correlations of Prototype 2 of the peritraumatic reactions instrument in the São Paulo and Rio de Janeiro, Brazil, samples.

	São Paulo			Rio de Janeiro		
$\sqrt{{ave}_{\left(f1\right)}}$	$\sqrt{{ave}_{\left(f2\right)}}$	$\sqrt{{ave}_{\left(f3\right)}}$	$\sqrt{{ave}_{\left(f1\right)}}$	$\sqrt{{ave}_{\left(f2\right)}}$	$\sqrt{{ave}_{\left(f3\right)}}$
$\emptyset_{f_{\left(1\right)}↔f_{\left(2\right)}}$	0.048 *	0.100 **		0.002	0.038 **	
$\emptyset_{f_{\left(1\right)}↔f_{\left(3\right)}}$	0.057 *		0.086 **	0.033		0.076 **
$\emptyset_{f_{\left(2\right)}↔f_{\left(3\right)}}$		0.130 **	0.107 **		0.103 **	0.109 **

AVE: average variance extracted.

* p < 0.05;

** p < 0.001.

Box 2 presents Prototype 3, with items outlined by their respective dimensions and scales following the original proposals. This prototype is also presented in a Brazilian Portuguese version as Supplementary Material (https://cadernos.ensp.fiocruz.br/static//arquivo/supl-e00062225_1885.pdf).

**Box 2:** The proposed *Peritraumatic Response Questionnaire*.

TONIC IMMOBILITY	SEVERITY RATING						
Rate the degree to which you froze or felt paralyzed during your most recent experience	0	1	2	3	4	5	6
	Not at all						Extremely/Very much
Rate the degree to which you were unable to move even though not restrained	0	1	2	3	4	5	6
	Not at all						Extremely/Very much
Rate the degree to which your body was trembling/shaking during the event	0	1	2	3	4	5	6
	Not at all						Extremely/Very much
Rate the degree to which you were unable to call out or scream during the event	0	1	2	3	4	5	6
	Not at all						Extremely/Very much
Rate the degree to which you felt numb or in no pain during the event	0	1	2	3	4	5	6
	Not at all						Extremely/Very much
Rate the extent to which you felt fear/panic during the event	0	1	2	3	4	5	6
	Not at all						Extremely/Very much
Rate the extent to which you feared for your life or felt as though you were going to die	0	1	2	3	4	5	6
	Not at all						Extremely/Very much
Tell us how much you felt unable to escape even though you wanted to	0	1	2	3	4	5	6
	Not at all						Extremely/Very much
DISSOCIATION	SEVERITY RATING						
I had moments of losing track of what was going on - I “blanked out” or “spaced out” or in some way felt that I was not part of what was going on	0	1		2		3	4
	Not at all true	Slightly true		Somewhat true		Very true	Extremely true
My sense of time changed - things seemed to be happening in slow motion	0	1		2		3	4
	Not at all true	Slightly true		Somewhat true		Very true	Extremely true
What was happening seemed unreal to me, like I was in a dream or watching a movie or play	0	1		2		3	4
	Not at all true	Slightly true		Somewhat true		Very true	Extremely true
I felt as though I were a spectator watching what was happening to me, as if I were floating above the scene or observing it as an outsider	0	1		2		3	4
	Not at all true	Slightly true		Somewhat true		Very true	Extremely true
There were moments when my sense of my own body seemed distorted or changed. I felt disconnected from my own body or that it was unusually large or small	0	1		2		3	4
	Not at all true	Slightly true		Somewhat true		Very true	Extremely true
I felt as though things that were actually happening to others were happening to me, like I was being trapped when I really was not	0	1		2		3	4
	Not at all true	Slightly true		Somewhat true		Very true	Extremely true
I was surprised to find out afterward that a lot of things had happened at the time that I was not aware of, especially things I ordinarily would have noticed	0	1		2		3	4
	Not at all true	Slightly true		Somewhat true		Very true	Extremely true
I felt confused; that is, there were moments when I had difficulty making sense of what was happening	0	1		2		3	4
	Not at all true	Slightly true		Somewhat true		Very true	Extremely true
I felt disoriented; that is, there were moments when I felt uncertain about where I was or what time it was	0	1		2		3	4
	Not at all true	Slightly true		Somewhat true		Very true	Extremely true
I felt “numbed” or emotionless	0	1		2		3	4
	Not at all true	Slightly true		Somewhat true		Very true	Extremely true
PHYSICAL PANIC REACTIONS	SEVERITY RATING						
Sensations of shortness of breath or smothering	0		1		2		3
	No		A little		Moderate		Extreme
Feeling dizzy, unstead, lightheaded, or faint	0		1		2		3
	No		A little		Moderate		Extreme
Palpitations, pounding heart, or accelerated heart rate	0		1		2		3
	No		A little		Moderate		Extreme
Trembling or shaking	0		1		2		3
	No		A little		Moderate		Extreme
Sweating	0		1		2		3
	No		A little		Moderate		Extreme
Nause or abdominal distress	0		1		2		3
	No		A little		Moderate		Extreme
Paresthesias (numbness or tingling sensations)	0		1		2		3
	No		A little		Moderate		Extreme
Chills or hot flushes	0		1		2		3
	No		A little		Moderate		Extreme
Feeling of choking	0		1		2		3
	No		A little		Moderate		Extreme
Chest pain or discomfort	0		1		2		3
	No		A little		Moderate		Extreme
Difficulty controlling bladder or bowel	0		1		2		3
	No		A little		Moderate		Extreme

## Discussion

The third and final prototype (Prototype 3) successfully met all evaluated configurational and metric properties. It comprises 29 items distributed across three dimensions: eight in the peritraumatic tonic immobility dimension, 10 in the peritraumatic dissociation dimension, and 11 in the peritraumatic physical reactions dimension. The instrument holds theoretical-empirical congruence, with all items uniquely associated with their respective factors. Moreover, modification indices and expected parameter changes analyses in the CFA confirmed factorial specificity, as no cross-loadings were observed. Evaluation of the metric structure shows strong item reliability, with high factor loadings and low residuals. The analyses also suggested an absence of content redundancy, since no significant residual correlations were detected. Both factor-based convergent validity and factor-based discriminant validity were fully supported.

The initial qualitative evaluation (step 1) with experts addressing the construct and its three underlying dimensions led to the relocation of some items from the original empirical scales. As aforementioned, along with the 30 items from the original instruments, experts proposed adding six new ones to the dataset of the original project, selected for their relevance to peritraumatic experiences and responses in the defensive cascade model (3). However, the qualitative stage ultimately excluded two items (res_it2: remembering details, and res_it3: feeling guilt/shame). Although these items are somewhat related to later symptoms of traumatic exposure ^29^ ^,^ ^30^, the experts concluded that remembering details and feeling guilt or shame were not manifestations occurring at the time of the trauma and thus not representative of peritraumatic reactions per se.

The remaining four external items were distributed across the three dimensions. Since the inability to move or scream without losing consciousness is considered a central element of the tonic immobility reaction in situations such as sexual assault or life-threatening experiences, res_it1 (unable to escape) was allocated to the PTI dimension. The items res_ppr1 (loss of bladder/bowel control) and res_ppr2 (fainting) were added to the PPR dimension, as both tend to occur during intense fear in response to parasympathetic system activations ^3^ ^,^ ^31^ ^,^ ^32^. Lastly, res_pd1 (numbed) was assigned to the peritraumatic dissociation dimension, as emotional numbness is part of a broader range of symptoms associated with altered states of consciousness - including emotional detachment, out-of-body experiences, depersonalization, and derealization - reflecting dissociation ^3^.

Based on the previously described concept of dissociation, we decided to reassign items tis9 (detached from self) and tis10 (distant from the situation) from the peritraumatic tonic immobility to the peritraumatic dissociation dimension. Since tremors and cold/shivering have been described as peritraumatic tonic immobility and physical reactions ^3^ ^,^ ^31^, we conservatively kept items tis3, tis6, ppr4, and ppr8 in the dimensions suggested by their original scales. Their dimensional pertinence, reliability, and non-redundancy were subsequently evaluated in later analytical steps.

Items tis7 (fear or panic) and tis8 (thought would die) were retained within the peritraumatic tonic immobility dimension. While these items could be organized under the peritraumatic physical reactions dimension, they indicated extreme risk perception during the traumatic event. Studies suggest that tonic immobility is more prevalent in situations of intense fear, high perceived threat, and contexts involving physical restraint or a sense of confinement ^18^ ^,^ ^31^. Notably, these two items are not strictly physical reactions commonly associated with panic attacks that occur immediately after a traumatic event.

In the second stage, we explored the configural structure of Prototype 1. Despite finding four eigenvalues above 1 in a preliminary PCA, we opted for a trifactorial solution. This decision was informed by subsequent ESEM analysis, in which the fourth factor showed low loadings (below 0.3) for most items. Three items (tis3, ppr3, and ppr4) with borderline loadings (0.3-0.5) did not form a theoretically cohesive set justifying a fourth factor (Box 1). The four-factor solution also yielded multiple cross-loadings, denoting insufficient specificity concerning a singular target factor.

During the second stage of our analysis, we encountered theoretical and factorial pertinence issues with certain items when examining their distribution across the three ESEM-derived factors. Out of the four items initially developed for the background study and retained in the qualitative stage, three were kept in Prototype 2: res_it1 (unable to escape), res_pd1 (numbed), and res_ppr1 (bladder or bowel control). Item res_ppr2 (fainting) was excluded due to borderline reliability. This decision was not considered problematic, as ppr2 (dizziness/fainting sensation) still captured the substantive manifestation of the peritraumatic physical reactions dimension. Furthermore, the modification indices suggested residual correlation found in the interim CFA backed a content redundancy involving these two items, thus reinforcing our decision.

Four items from the original scales used in constructing the prototype were eliminated. The item pdeq2 (“autopilot”) was excluded due to low reliability, consistent with previous studies examining the PDEQ ^33^ ^,^ ^34^ ^,^ ^35^. Item tis6 (feeling cold) was also excluded despite borderline reliability, with minimal overall loss, as the underlying manifestation was likely captured by ppr8 (chills and hot flushes). Initially assigned to the peritraumatic dissociation dimension during the qualitative stage, items tis9 (disconnected from oneself) and tis10 (distant from the situation) were ultimately excluded due to their cross-loadings in the peritraumatic tonic immobility and peritraumatic dissociation factors. The non-specificity regarding the peritraumatic tonic immobility factor could be attributed to a process effect, as these items were applied immediately after other TIS items. Regardless, we are confident that the peritraumatic dissociation dimension mapping was not compromised, as a content-equivalent item, pdeq6 (altered body), was retained in Prototype 3.

Three items from the TIS - tis3 (trembling or shaking), tis7 (fear or panic), and tis8 (thinking you were going to die) - offered acceptable reliability regarding their hypothesized peritraumatic tonic immobility dimension. However, they also displayed borderline loadings on the PPR factor, which was expected given the overlap between peritraumatic tonic immobility and certain panic-like manifestations reported in PTSD literature ^31^. Based on theoretical considerations, we decided to maintain these items in their original dimension for further evaluation. In the ensuing CFA, all three items exhibited strong reliability (above 0.7) in the peritraumatic tonic immobility dimension, sustaining their inclusion in Prototype 3.

Several aspects of the study warrant emphasis. On a positive note, data were derived from extensive, independent, and representative samples of the general population of two major cities. This enabled complementary analyses in very similar linguistic and sociocultural contexts, thus enhancing the consistency of the findings. Furthermore, specific procedures were employed to ensure data quality during data collection. The field study was conducted in a suitable operational setting (households) and supervised by a company experienced in large-scale population studies. Moreover, data collection teams in both cities were trained by the same researcher, contributing to methodological consistency.

However, caution is warranted, as data was not primarily collected to develop a peritraumatic reaction instrument. Consequently, we could not address any problematic items identified in the psychometric analyses by revising question formats or response options, nor by substituting them with newly designed items. The post hoc nature of this study also precluded us from altering the sequence of items in the multi-thematic questionnaire of the main study. These limitations should be explored in future research, building upon the “final” prototype presented here.

Further refinements are also required, including examining measurement invariance across sociodemographic groups and geographically defined strata, as done in our study ^36^. Although robust evidence is presented here, our results are limited to the Portuguese version adapted for use in Brazil. To enhance the applicability and legitimacy of the instrument, psychometric evaluations of Prototype 3 (or an offshoot) should be extended to other sociolinguistic and cultural contexts. Additionally, investigating scalability properties would inform the standardization of response categories for each item ^37^. Finally, it is essential to incorporate studies assessing external validity via hypothesis testing for the ongoing refinement of the instrument ^38^.

This study aimed to harmonize items from three existing instruments alongside standalone items to propose a comprehensive tool for measuring peritraumatic reactions. A notable advantage of a single measurement device encompassing the three core dimensions is avoiding item overlap that may occur when employing specific instruments. Importantly, this research explores how different types of peritraumatic reaction affect physical and mental health, regarding both their individual impact and co-occurrence.

By integrating qualitative and quantitative approaches, we successfully obtained a final set of 29 items organized into three dimensions, demonstrating robust configural and metric properties. While validation is still ongoing, the instrument may be already suitable for epidemiological studies. Cautiously, we provisionally recommend its use as the *Peritraumatic Response Questionnaire* (PTRQ). Future studies can further refine the instrument, enhancing its comprehensiveness and broader applicability.

## Data Availability

The research data are available upon request to the corresponding author.
