# Supplementary material for: Development and validation of a comprehensive tool for measuring multidimensional peritraumatic experience
Source: Cad Saude Publica. 2025 Dec 1;41(11):e00062225. doi: 10.1590/0102-311XEN062225 (PMC12688308; doi:10.1590/0102-311XEN062225)
Supplement: Supplementary Material [file 1678-4464-csp-41-11-EN062225-s.pdf]

Supplementary Material: Portuguese version of the proposed *Peritraumatic Response Questionnaire.*

**IMOBILIDADE TÔNICA**	**INTENSIDADE DAS REAÇÕES**						
Diga o quanto você se sentiu congelado ou paralisado durante o acontecimento	0	1	2	3	4	5	6
	Não me senti paralisado ou congelado						Senti-me totalmente paralisado ou congelado
Diga o quanto você se sentiu incapaz de se mexer mesmo que ninguém o estivesse prendendo durante o acontecimento	0	1	2	3	4	5	6
	Senti-me capaz de me mexer						Senti vontade de me mexer, mas fiquei “travado”
Diga o quanto seu corpo ficou tremendo ou sacudindo durante o acontecimento	0	1	2	3	4	5	6
	Não fiquei tremendo ou sacudindo						Fiquei tremendo ou sacudindo extremamente
Diga o quanto você se sentiu incapaz de gritar mesmo tendo vontade durante o acontecimento.	0	1	2	3	4	5	6
	Senti-me capaz de gritar						Senti vontade de gritar, mas fiquei “travado”
Diga o quanto você se sentiu anestesiado ou sem dor durante o acontecimento.	0	1	2	3	4	5	6
	Não me senti anestesiado						Extremamente anestesiado
Diga o quanto você sentiu medo ou pânico durante o acontecimento.	0	1	2	3	4	5	6
	Fiquei totalmente calmo						Senti medo extremo
Diga o quanto você achou que ia morrer durante o acontecimento.	0	1	2	3	4	5	6
	Não achei que ia morrer						Tive certeza absoluta de que ia morrer
Diga o quanto você se sentiu incapaz de escapar mesmo tendo vontade	0	1	2	3	4	5	6
	Senti-me capaz de escapar						Senti vontade de escapar, mas fiquei “travado”

**DISSOCIAÇÃO**	**INTENSIDADE DAS REAÇÕES**				
Houve momentos em que eu perdi a noção do que estava acontecendo – “Me deu um branco” ou “eu saí do ar” ou de alguma forma eu senti como se eu não fizesse parte do que estava acontecendo	0	1	2	3	4
	Não	Leve	Moderado	Muito	Extremamente
Minha noção do tempo mudou – as coisas pareciam estar acontecendo em câmera lenta	0	1	2	3	4
	Não	Leve	Moderado	Muito	Extremamente
O que estava acontecendo parecia que não era real, como se eu estivesse num sonho ou assistindo um filme ou uma peça de teatro	0	1	2	3	4
	Não	Leve	Moderado	Muito	Extremamente
Eu senti como se estivesse assistindo tudo o que estava acontecendo comigo pelo lado de fora, como um espectador, ou como se eu estivesse flutuando, vendo tudo de cima	0	1	2	3	4
	Não	Leve	Moderado	Muito	Extremamente
Houve momentos em que a noção que eu tinha do meu próprio corpo parecia distorcida ou modificada. Eu me senti desligado do meu corpo ou que meu corpo estava maior ou menor do que o habitual	0	1	2	3	4
	Não	Leve	Moderado	Muito	Extremamente
Eu senti como se as coisas que estavam acontecendo com outras pessoas estivessem acontecendo comigo – por exemplo, alguém foi preso, e eu senti que era eu quem estava sendo preso	0	1	2	3	4
	Não	Leve	Moderado	Muito	Extremamente
Eu fiquei surpreso por descobrir mais tarde que várias coisas que tinham acontecido naquela ocasião eu não havia percebido, principalmente coisas que eu normalmente teria notado	0	1	2	3	4
	Não	Leve	Moderado	Muito	Extremamente
	Não	Leve	Moderado	Muito	Extremamente
Eu me senti confuso: ou seja, houve momentos em que eu tive dificuldade para entender o que estava acontecendo	0	1	2	3	4
	Não	Leve	Moderado	Muito	Extremamente
Eu me senti desorientado: ou seja, houve momentos em que eu me senti perdido no tempo e no espaço	0	1	2	3	4
	Não	Leve	Moderado	Muito	Extremamente
Eu me senti “entorpecido” ou sem emoção	0	1	2	3	4
	Não	Leve	Moderado	Muito	Extremamente

**REAÇÕES FÍSICAS DE PÂNICO**	**INTENSIDADE DAS REAÇÕES**

Falta de ar	0	1	2	3
	Nada	Um pouco	Moderadamente	Extremamente
Tonteira ou sensação de desmaio	0	1	2	3
	Nada	Um pouco	Moderadamente	Extremamente
Coração acelerado	0	1	2	3
	Nada	Um pouco	Moderadamente	Extremamente
Tremores ou abalos	0	1	2	3
	Nada	Um pouco	Moderadamente	Extremamente
Sudorese (suor intenso)	0	1	2	3
	Nada	Um pouco	Moderadamente	Extremamente
Náuse ou desconforto abdominal	0	1	2	3
	Nada	Um pouco	Moderadamente	Extremamente
Dormência no corpo ou sensação de formigamento	0	1	2	3
	Nada	Um pouco	Moderadamente	Extremamente
Ondas de calor ou calafrios	0	1	2	3
	Nada	Um pouco	Moderadamente	Extremamente
Sufocação	0	1	2	3
	Nada	Um pouco	Moderadamente	Extremamente
Dor ou desconforto no peito	0	1	2	3
	Nada	Um pouco	Moderadamente	Extremamente
Dificuldade para controlar a bexiga ou intestino	0	1	2	3
	Nada	Um pouco	Moderadamente	Extremamente
